# Diallel analysis of common bean (*Phaseolus vulgaris* L.) genotypes for seed dietary fibre, carbohydrate, calcium and phosphorus contents

**DOI:** 10.1007/s13353-024-00834-7

**Published:** 2024-02-14

**Authors:** Aladji Abatchoua Madi Madi Ibram, Yadji Haman Taidi, Likeng Li Ngue Benoit-Constant, Noubissié Tchiagam Jean-Baptiste, Ibrahima Adamou

**Affiliations:** 1 Department of Sciences and Technics of Biological Agriculture, Faculty of Science, University of Ngaoundéré, P.O. Box 454, Ngaoundéré, Cameroon; 2 Department of Biological Sciences, Faculty of Science, University of Ngaoundéré, P.O. Box 454, Ngaoundéré, Cameroon; 3 Genetic and Plant Breeding Unity, Department of Plant Biology, Faculty of Science, University of Yaoundé1, , P.O. Box 812, Yaounde, Cameroon

**Keywords:** *Phaseolus vulgaris*, Genetic information, Breeding strategies, Bean seed traits, Guinean Savannah zone

## Abstract

Genetic information of bean seed traits can be an immense help to the breeder in selection of suitable genotypes and the appropriate breeding strategies. Therefore, the investigation aims to assess the genetic variability and to elucidate the genetic analysis of seed dietary fibre, carbohydrate, seed calcium and phosphorus contents of *Phaseolus vulgaris* in the high Guinean Savannah zone conditions. 5 × 5 half-diallel crosses of these traits were conducted in randomized complete block design with three replications. Results revealed high differences between five lines beans (*p* < 0.05), suggesting the sufficient genetic diversity for these traits. High broad sense heritability values were recorded for seed dietary fibre, carbohydrate and seed calcium content, attesting a strong implication of the genetic factors in the control of these traits; thereby, these traits can be improved through regular selection. The ratio GCA/SCA was greater than unity only for seed phosphorus content. It indicates the prevalence of additive gene effect in the involvement of the genetic control for this trait. The combining ability analysis revealed highly significant differences between parental GCA effects and F_1_ cross SCA effects. The PB, BI, CT and PR lines beans will prove useful in common bean breeding programmes as donor genotypes, in the development of bean genetic resources for betterment improvement of nutritional traits.

## Introduction

Common bean (*Phaseolus vulgaris* L.) is one of the most important legume crops used for direct human consumption. Dry bean is a short-season leguminous crop capable of hosting rhizobia responsible for biological nitrogen fixation (Reinprecht et al. 2020). The dry bean supplies food security, and nutritional value was linked in sub-Saharan Africa, Latin America and the Caribbean (Joshi and Rao 2016). It is an important source of protein, fibre, carbohydrates, vitamins and minerals and represents nearly half of the consumed grain legumes worldwide (Duc et al. 2015). Due to their high protein, mineral and fibre content, beans are consumed instead of meat in underdeveloped and developing countries (Reyes-Moreno and Paredes-Lopez 1993). The dietary fibre contributes to reducing morbidity and mortality associated with chronic diseases that currently account for > 70% of deaths globally per annum with the total amount of dietary fibre being one predictive benefit (Thompson and Brick 2016). In fact, numerous health benefits have been associated with consuming adequate amounts of dietary fibre including lower blood cholesterol, reduced risk of heart disease, increased faecal bulk, decreased intestinal transit time, reduced risk of colon cancer and improved glucose tolerance which is especially beneficial for diabetics (Schneeman 1986). Additionally, dietary fibres are important due to its high content of metabolically active soluble dietary fibre and its effectiveness in lowering blood cholesterol (Anderson et al. 1984). Carbohydrate is the first dominant part of proximate composition that has a great role in providing good source of energy (Aremu et al. 2009). *P. vulgaris* contents micronutrients, such as phosphorus (P) and calcium (Ca) are essential minerals for both human diet and provide vital functions in plants and are required in large quantities (White and Broadley 2009). Among minerals, calcium stands out due to its importance in bone and tooth formation, as well as its role in growth and its being a co-factor/regulator in various biochemical reactions (Miglioranza et al. 2003). Further, beans are an excellent dietary alternative to dairy products since they contain a high quantity of calcium (Frossard et al. 2000). Calcium intervened in the maintenance of the mineral homeostasis and physiological performance in general (Morgan 2008). According to Heppler and Wayne (1985), calcium has been described as mineral for the maintenance of cell membranes and walls because it takes part in links with pectin substances which help to cell adhesion. From the pointed of view of Yang and Finnegan (2010), phosphorus is crucial in energy metabolism and photosynthesis, biosynthesis of organic compounds, the structural element of nucleic acids and phospholipids, up- and downregulation of gene and the activity of enzymes, and also in signal transduction. Moreover, deficiency in phosphorus is uncommon but symptoms are described as painful bones, irregular breathing, fatigue, anxiety, numbness, skin sensitivity and changes in body weight (Ghosh and Joshi 2008).

Despite the importance of bean seed nutritional value, information on the inheritance of the seed dietary fibre and total carbohydrate is insufficient, singularly in the current genetic resources available in Cameroon. “Developing new common bean varieties with considerable seed dietary fibre, carbohydrate and the mineral contents are vital for effective selection”. “Exploitation of genetic variability is a pre-requisite for the effective screening of superior lines bean”. Thus, Ceyhan et al. (2014), Chen et al. (2016), and Sahasakul et al. (2022) reported a wide genetic variability for dietary fibre, carbohydrate and mineral elements in bean seeds. Previous studies showed that seed dietary fibre and carbohydrate were inherited (Patrícia et al. 2006; Jemal et al. 2022; Kevin et al. 2022). “Moreover, to achieve knowledge on the heredity of a trait, several mating schemes are available, including diallel crosses”. Ceyhan et al. (2014) and Gami et al. (2020) asserted that diallel crosses are the fastest method to understand the heredity of quantitatively traits and find out the predominance of parents. “Thus, estimation of general combining ability (GCA) of each parent would contribute to develop highly potential genotypes with specific combining ability (SCA) effect in order to evaluate the hybrid performances in diallel crosses”. Therefore, the present study aims to assess the variability and to clarify genetic analysis of seed dietary fibre, carbohydrate, calcium and phosphorus contents in *Phaseolus vulgaris* through 5 × 5 half-diallel mating design under the high Guinean Savannah zone conditions.

## Materials and methods

### Site

The investigation was carried out from 2017 to 2018 at the University of Ngaoundéré, on an experimental site, at Dang locality (Ngaoundéré 3rd subdivision, Adamawa region, Cameroon), which intersected “7° 26 16 4’’ North Latitude and “13° 33′ 34’’ East Longitude and has 1115 m above the mean sea level. This region belongs to the Guinean High Savannah agro-ecological zone (Djoufack et al. 2012). The climate is of the Sudano-Guinean type distinguished with a humid trend, an average annual rainfall of 1480 mm given out over the rainy season (March–October) and dry season (November–March). The average annual temperature is 22.59 °C, while the relative humidity is about 66.47%. The soil in the area is mainly ferruginous type developed on old basalt and has a brown reddish clay texture. The pH of the soil varies between 5.5 and 6. There is a huge dependence of agriculture productivity for soil physicochemical properties (Nanganoa et al. 2020). The soil physicochemical properties for experimental site are presented in Table 1.

**Table 1:** Characteristics of physicochemical soil properties for experimental site

Genotypes	pH	Phosphorus (mg kg^−1^)	Calcium (mg kg^−1^)
Zizi fho meko (PB)	5.75	485 ± 0.44	217.66 ± 0.15
Meko Atsa (PH 114/CT)	5.50	470 ± 0.35	222.66 ± 0.21
Pan Magreta (PH 181/BI)	5.98	567 ± 1.00	232.33 ± 0.65
She Menko (PH 460/PN)	5.66	459.66 ± 0.28	204.00 ± 1.00
Merengue (PH 123/PR)	5.59	567.16 ± 0.25	201.33 ± 0.78

### Samples

Breeding material comprised of five lines beans genetically diversified are chosen based on their adoption rate by growers in the Grassfields zone of Cameroon (Noubissié et al. 2000) and were provided by the Institute of Agricultural Research for Development (IRAD, Dschang station, West Region, Cameroon) through Bean programme, namely, Zizi fho meko (PB), Meko Atsa (PH 114/CT), Pan Magreta (PH 181/BI), She Menko (PH 460/PN) and Merengue (PH 123/PR) (Table 2).

**Table 2:** Characteristics of five lines beans

Genotypes	Date	Growing area	Seed coat colour	Seed index	Cooking time (min)
Zizi fho meko (PB)	1960	West region	White	40.33	52.55
Meko Atsa (PH 114/CT)	Local	Menoua	Maroon	19.16	73.92
Pan Magreta (PH 181/BI)	Local	Grassfields	Brown spotted	17.89	57.66
She Menko (PH 460/PN)	1960	West region	Black	31.26	89.21
Merengue (PH 123/PR)	1960	West region	Red speckled	18.00	49.28

Date: Date of introduction in Cameroon; local varieties are those introduced during the colonial period.

An initial field trial was undertaken in 2017, growing at the rainy season in order to assess the genetic variability of common bean for seed dietary fibre, carbohydrate, calcium and phosphorus contents. Five lines beans were sown in pots from April to June 2017 for emasculation and doing crosses between lines bean. The 5 × 5 half-diallel scheme was used to develop sets of 10 F_1_ hybrids [*n* (*n* − 1)/2]. No fertilizer and plant protection treatment were used in this study. Mature pods were progressively harvested, and healthy seeds were carefully removed and kept in tagged envelopes.

### Field trials

During the 2018 rainy season, all the five lines and the ten F_1_ hybrids were conducted in randomized complete block design (RCBD) with three replications. Each plot unit consisted of one row of 4 m length × 0.5 m broad, spaced out 40 cm apart. The distance between blocks was 1 m and 0.5 m between experimental units. Each experimental unit is 2-m long and 1.5-m wide. The bean seeds were sown at an intra-row spacing of 20 cm. Three seeds were dibbed per hole, and after seed germination, one healthy seedling was held at each hole after thinning, 20 days after sowing (DAS). The plots were manually weeded at 20 DAS, 40 DAS and 60 DAS. At anthesis, plant to plant pollination of all possible crosses except reciprocals, was made following the 5 × 5 diallel crossing pattern. Each cross was tagged for easy identification, and, at maturity, the dry bean seeds were harvested separately, and they were ground to powder using a Culatti hammer mill (Polymix, France) through a 500-µm mesh sieve for nutritional component analysis.

### Experimental measurements

#### Determination of seed dietary fibre

Dietary fibre was evaluated according to Prosky et al. (1988) method. 0.255 N of sulphuric acid was added to 1 g of bean line sample. The mixture was boiled for 30 min in a water bath and centrifuged. 0.313 of sodium hydroxide was added to residue and boiled for 30 min. The residue obtained was washed three times with hot distilled water and then twice with acetone. The insoluble material obtained was dried at 105 °C for 8 h and was weighed (m1). The residue was subjected to incineration at 550 °C for 3 h in a muffle furnace, and the ashes were weighed (m2).

The crude fibre content (g/100 g DW) was given in the following formula:

$$F=\left(m1-m2\right)\times 100/m\times (100-TE)$$

where *m* is the mass of sample (g), m1 is the mass of residue after steaming (g), m2 is the mass of residue after incineration (g) and TE is the residual water content of sample.

#### Determination of seed carbohydrate

Carbohydrate was estimated using the colorimetric method described by Dubois et al. (1956). Five millilitres of 1.5 N of acid sulphuric was added to the 0.2 g of line bean sample. The mixture was heated in a water bath at 100 °C for 45 min and then cooled to a room temperature. Ten millilitres of 70% ethanol, 1 ml of zinc acetate (2 g/100 ml) and 1 ml of potassium ferrocyanide (10.6 g/100 ml) were added for defecation. The mixture was filtered into a 50-ml flask, and then, the filtrate volume was made up to 50 ml with distilled water. Using a 1 mg/ml glucose standard solution, prepare the range.

The carbohydrate in the test portion was determined by plotting the regression equation:

$$DO=aQ+b$$

The total sugar estimated was expressed in g/100 g dry weight seed (DW) following the formula:

$${{\text{Q}}}^{\mathrm{^{\prime}}}=100\times \frac{QVT}{m.V}\times ( 100-H^\circ r)$$

where *Q* is the sugar quantity in the test portion, VT is the total volume of extract, *m* is the mass sample (g), *V* is the volume of sample, and Hr is the the residual water content.

#### Calcium and phosphorus analysis

The analysis of calcium (Ca) was performed according to the method proposed by Benton and Vernon (1990). 0.1 g of ash bean seeds, obtained by incineration at 550 °C in a muffle furnace of bean seed powders, after steaming at 105 °C, was dissolved in 4 ml of concentrated hydrochloric acid, and the solution was filtered into a 10-ml flask. The volume was made up with distilled water before the calcium content was determined by atomic absorption spectroscopy.

Phosphorus content was analysed according to the method given by Murphy and Riley (1962). One gramme was treated with 10 ml of hydrochloric acid and then was made up with 100 ml of distilled water. Reagent combined was prepared by first mixing the 10 ml of 50% sulphuric acid and was made up with 50 ml of water. After cooling the mixture in order to avoid heating due to the dissolution of the sulphuric acid, 1 g of ammonium molybdate and 40 mg of potassium antimony double tartrate were added. The volume was adjusted with 100 ml of distilled water. After preparing 100 ml of 20 g/l ascorbic acid solution, 1 g of seed bean powders of each variety was mixed with the combined reagent and ascorbic acid. After 15 mn, the mixture was filtered through a Wattman paper, and the optical densities (OD) were read at 885 nm.

### Statistical analyses

All nutritional components were done in three replications. For the genetic variability, data obtained from the five lines beans were subjected to analysis of variance (ANOVA) using STATGRAPHICS PLUS 15.1 software, and mean significant differences were tested by the least significant difference (LSD) at the 5% level.

### Diallel analysis

The diallel analysis was done using DIAL 98 software (Ukai 2002). The Griffing (1956) method 2 (excluding reciprocal F_1_ crosses), model 1 (fixed effects) was used to evaluate the general combining ability of parents (parents ability to combine among each other during hybridization process such that desirable genes or characters are transmitted to their progenies) and the specific combining ability of hybrid F_1_ (the deviation of hybrid combination performance from the performance expected on the basis of the general combining ability of the parental inbred lines), amplified by the analysis of variance by Walters and Morton (1978). With this approach, the sources of variation were partitioned into the additive effects (a) and the dominance effects (b) which were further sub-divided into b_1_, b_2_ and b_3_. The genetic parameters were calculated as per Hayman (1954). Broad sense heritability (*h*^2^) was measured as the proportion of genetic variance ($\delta$ ^2^ g) in the phenotypic variance between the parents ($\delta$ ^2^p), while narrow sense heritability (*h*^2^_n_) was evaluated as the proportion of additive variance ($\delta$ ^2^_A_) in the phenotypic variance between the parents ($\delta$ ^2^p) (Mather and Jinks 1982). The simple additive-dominance model was tested by the regression of the covariance values between the parents and their offspring in the r^th^ array (Wr) against variance values of the r^th^ array (Vr).

## Results

Table 3 shows the mean values for seed dietary fibre, carbohydrate, calcium and phosphorus content of five common beans. Highly significant differences at 5% probability levels (*p* < 0.01) were observed in this germplasm for these traits. The values ranged from 20.50 to 23.84 g/100 g DW for seed dietary fibre while carbohydrate values varied from 45.70 to 50.53 g/100 g DW. The highest dietary fibre was found in the PB genotype (23.84 ± 0.26 g/100 g DW), and the lowest value was recorded by PR genotype (20.50 ± 0.26 g/100 g DW). PR line had a high carbohydrate (50.53 ± 0.37 g/100 g DW) than BI line (44.53 ± 0.37 g/100 g DW). Among the genotypes, BI variety showed the highest calcium content (118.33 ± 0.57 mg/100 g DW) while PR genotype had the strongest phosphorus content (347.66 ± 0.57 mg/100 g DW) of bean seeds.

**Table 3:** Variability of seed dietary fibre, carbohydrate, calcium and phosphorus contents in five lines beans

Genotypes	Dietary fibre (g/100 g DW)	Carbohydrate (g/100 g DW)	Calcium (Ca) (mg/100 g DW)	Phosphorus (P) (mg/100 g DW)
Zizi fho meko (PB)	23.84 ± 0.26^a^	49.26 ± 0.44^b^	110.66 ± 0.57^c^	330 ± 0.57^c^
Meko Atsa (PH 114/CT)	23.26 ± 0.15^b^	45.70 ± 0.10^d^	112.66 ± 0.57^b^	330 ± 0.57^c^
Pan Magreta (PH 181/BI)	23.46 ± 0.25^ab^	44.53 ± 0.37^e^	118.33 ± 0.57^a^	341 ± 1.00^b^
She Menko (PH 460/PN)	22.43 ± 0.30^c^	47.66 ± 0.20^c^	98.00 ± 1.00^d^	340.66 ± 0.57^b^
Merengue (PH 123/PR)	20.50 ± 0.26^d^	50.53 ± 0.37^a^	96.33 ± 0.57^e^	347.66 ± 0.57^a^
Mean	22.69	47.53	107.19	337.86
Least significant different	10.03	1.6	2.00	10.66

Means of five lines bean values for each trait with the same subscript within the same column do not differ significantly only at 5%

Referring to Griffing (1956) method, Table 4 presents the analysis of variance which revealed significant effects (*p* < 0.01) of mean squares of general and specific combining abilities for seed dietary fibre, carbohydrate, calcium and phosphorus contents in dry bean seeds. Mean squares were significantly different among these traits. The variance due to GCA was higher than that of SCA only for seed phosphorus content.

**Table 4:** Mean squares for general and specific combining abilities in 5 × 5 half-diallel crosses for seed dietary fibre, carbohydrate, calcium and phosphorus contents in the dry bean seeds

Source of variation	Degree of freedom	Mean squares			
		Dietary fibre	Carbohydrate	Calcium	Phosphorus
GCA	4	4.89**	7.81*	3041.73**	475813.00**
SCA	5	0.29*	2.53**	4839.40**	380641.00**
Error	38	0.16	0.02	0.13	0.22
*δ*^2^ GCA/*δ*^2^ SCA		0.14	0.76	0.33	1.26

*GCA* general combining ability effects, *SCA* specific combining ability effects.

*Significant at *p* = 0.05.

**Significant at *p* = 0.01.

Using the ANOVA method of Walters and Morton (1978), it showed that both additive (a) and dominance (b) effects were all significant (*p* < 0.01) for seed dietary fibre, carbohydrate, calcium and phosphorus contents in common bean (Table 5). Within the dominance components (b_1_, b_2_ and b_3_), the mean dominance effects (b_1_) and the additional dominance effects due to the parents (b_2_) and residual dominance effects (b_3_) were highly significant (*p* < 0.01) for these traits excepted for dietary fibre in lines bean.

**Table 5:** Mean squares from analysis of variance, for additive and dominance effects of seed dietary fibre, carbohydrate, calcium and phosphorus contents in the dry bean seeds

Source of variation	Degree of freedom	Mean squares			
		Dietary fibre	Carbohydrate	Calcium	Phosphorus
Repetition	2	0.01^ ns^	0.21^ ns^	0.92^ ns^	1.86^ ns^
(a)	4	9.09**	22.45**	3.29*	270.98**
(b)	10	130.07**	609.60**	20.09**	285.69**
b_1_	1	1294.59**	6056.38**	437.00**	1817518**
b_2_	4	1.15**	6.74**	266.30**	851969**
b_3_	5	0.29*	2.54**	296.26**	1044508**
Error	28	0.15	0.18	0.11	0.14

*a*, additive effects of genes, *b*, dominant effects of genes, *b*_*1*_, mean dominance effects, *b*_*2*_, additional dominance deviation due to the parents, *b*_*3*_, residual dominance effects; *ns* indicates non significance at 5%

* and ** indicates significance at 5 and 1%, respectively.

Table 6 shows the estimated genetic components as well as heritability for seed dietary fibre, carbohydrate, calcium and phosphorus contents in common bean. For these traits, the dominance components H1 and H2 were significant and higher than those of component *D*. The lowest value of environmental variance (*E*) was recorded for seed dietary fibre, carbohydrate and phosphorus content. The estimated average degree of dominance (H1/*D*)^½^ was lesser than one, except seed phosphorus content. Furthermore, moderate values of proportion of dominant genes were observed for all traits. The high broad sense heritability values were noted excluding the seed phosphorus content, whereas the lowest narrow sense heritability values were obtained only for seed dietary fibre and carbohydrate in bean seeds.

**Table 6:** Estimated genetic components and heritability values for seed dietary fibre, carbohydrate, calcium and phosphorus contents in bean seeds

Estimated genetic components	Dietary fibre	Carbohydrate	Calcium	Phosphorus
Additive variance (*D*)	1.97*	7.82*	207.29*	56.65*
Dominance variance 1 (H1)	85.66**	394.83**	1399.84**	235.04**
Dominance variance 2 (H2)	85.44**	393.35**	1378.01**	214.06**
Environmental variance (*E*)	0.87	0.10	46.18*	0.12
Average degree of dominance (H_1_/*D*)^1/2^	6.59	7.10	2.59	0.31
Proportion of dominant genes kd/(kd + kr)	0.51	0.52	0.58	0.41
Broad sense heritability (*h*^2^)	0.99	0.99	0.89	0.69
Narrow sense heritability (*h*^2^_n_)	0.30	0.14	0.67	0.75

Table 7 presents the estimation a general combining ability effects (GCA) of five lines for dietary fibre, carbohydrate, calcium and phosphorus contents in bean seeds. Results revealed that the best desirable GCA effects for seed dietary fibre were found in PB and CT parents. PB and PR genotypes were highly exhibited with a significant positive GCA effects for seed carbohydrate in bean seeds. PB and BI lines showed a highest significant and positive GCA effects for calcium while PN and PR genotypes exhibited highly positive and significant GCA effects for phosphorus content.

**Table 7:** General combining ability effects (GCA) of dietary fibre, carbohydrate, calcium and phosphorus content in the dry bean seed varieties

Parents	General combining ability effects (GCA)			
	Dietary fibre	Carbohydrate	Calcium	Phosphorus
Zizi fho meko (PB)	0.55**	0.62**	1.73**	0.13
Meko Atsa (PH 114/CT)	0.54**	− 0.66	1.27*	1.13
Pan Magreta (PH 181/BI)	− 0.16*	− 0.55	3.38**	1.19
She Menko (PH 460/PN)	− 0.33*	0.23*	− 1.18	2.46**
Merengue (PH 123/PR)	− 0.60	0.35**	− 1.73	3.20**
Standard error (SE)	0.11	0.15	0.21	0.04

*Significant at *p* = 0.05

**Significant at *p* = 0.01

Table 8 presents the values of specific combining ability (SCA) effects of ten crosses for dietary fibre and carbohydrate of bean seed. Results revealed that PB × BI, CT × PN and BI × PR crosses expressed positive and highly significant specific combining ability effects for seed dietary fibre, while CT × BI cross had a desirable value of SCA effect for seed total carbohydrate. PB × PN, CT × PR, BI × PN and BI × PR crosses for calcium content had best SCA effects. PB × CT, CT × BI and CT × PN hybrids were found to be good specific combiners for phosphorus content in common bean.

**Table 8:** Specific combining ability effects (SCA) of dietary fibre, carbohydrate, calcium and phosphorus contents in the dry bean seed varieties

Crosses	Specific combining ability effects (SCA)			
	Dietary fibre	Carbohydrate	Calcium	Phosphorus
PB × CT	− 0.08	0.34*	− 1.08	3.00**
PB × BI	0.08**	0.34*	− 0.69	1.26*
PB × PN	0.06*	− 0.33	1.86**	1.33*
PB × PR	− 0.05	− 0.34	− 0.08	0.26*
CT × BI	− 0.07	0.35**	− 3.69	2.26**
CT × PN	0.27**	− 0.33	0.86*	1.99**
CT × PR	− 0.12	− 0.36	3.92**	0.26*
BI × PN	− 0.26*	− 0.36	2.75**	− 0.06
BI × PR	0.24**	− 0.33	1.64**	− 0.8
PN × PR	− 0.07*	1.03*	− 5.47	− 1.40
Standard error	0.10	0.30	0.15	0.13

Zizi fho meko (PB); Meko Atsa (PH 114/CT); Pan Magreta (PH 181/BI); She Menko (PH 460/PN); Merengue (PH 123/PR).

*Significant at *p* = 0.05.

**Significant at *p* = 0.01.

**Fig. 1:**
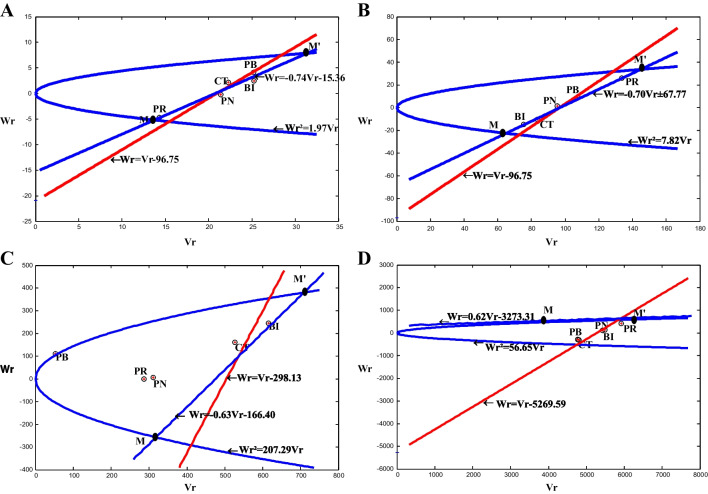
Wr/Vr graphs for seed dietary fibre (**A**), carbohydrate (**B**), calcium (**C**) and phosphorus contents (**D**). Wr.^2^ = VrVp: limiting parabola where Vp is the variance of the parents, Vr is the variance of the rth array and Wr is the covariance between the parents and their offspring in the rth array. Solid line: tangent to the limiting parabola (Wr = 1Vr + b); dotted line: regression of Wr on Vr. Zizi fho meko (PB); Meko Atsa (PH 114/CT); Pan Magreta (PH 181/BI); She Menko (PH 460/PN); Merengue (PH 123/PR)

The graphical presentation (Vr, Wr) for seed dietary fibre, carbohydrate and seed calcium content (Fig. 1) showed that the regression lines (Wr =  − 0.74Vr − 15.36; Wr =  − 0.70Vr ± 67.77; Wr =  − 0.63Vr − 166.40) intercepted the Wr-axis under the point of origin. In contrast, the regression line (Wr =  − 0.62Vr − 3273.31) cuts Wr-axis above the origin for seed phosphorus content.

## Discussion

Highly significant differences among the five common beans revealed the sufficient genetic diversity for seed dietary fibre, carbohydrate, calcium and phosphorus contents, showing a better possibility of improvement of these traits in bean seeds for diet enrichment through breeding strategies. Previous studies also have been done regarding genetic variability of dietary fibre in lentil (Khan et al. 2007), carbohydrate in *Phaseolus vulgaris* (Sahasakul Amornrat et al. 2022) and in cowpea (Garcia et al. 2010), seed calcium in cabbage (Singh et al. 2013) and for seed phosphorus contents in sorghum (Badigannavara et al. 2016). Evidently, the phosphorus content in bean seeds can be increased by applying fertilizer to the soil. Moraghan et al. (2002) asserted that the variation observed would be due to the seed-coat contains which was more than 80% of the entire calcium content and only ranged from 1.9 to 3.6% for the phosphorus content. According to Sahasakul et al. (2022), the highest of carbohydrate observed in dry bean seeds was due to the dietary fibre. As pointed out by Singh et al. (2004), the difference resulted to nitrogen application because if it applied and less than the amount of carbohydrate, it will increase. In addition, the availability of bean plants’ calcium depends on their concentration in the soil solution and also on chemical soil properties (Quintana et al. 1999). Previously, genetic diversity in common bean has been studied using different molecular markers such as allozymes (Singh et al. 1991; Santalla et al. 2002). Based on DArTseq-derived SNP markers, Carovic-Stanko et al. (2021) identified the quantitative trait nucleotides (QTNs) associated with the variation of seed bean phosphorus and calcium contents.

The GCA/SCA ratio was greater than one only for seed phosphorus content indicating the preponderance of additive gene effects involved in the control of this trait, and it must be improved through direct selection. Investigations doing by Fernandes and Boiteux (2015) in cowpea showed a quantitative inheritance as measured by additive-dominance model for seed calcium and phosphorus contents. According to Xavier et al. (2019), in Oka, dietary fibre is governed by additive gene effects. In contrast, Tamilselvi et al. (2015) showed that non-additive gene effects were involved in the heredity of seed carbohydrate in *Cucurbita moschata*, and it recommends hybrid production and heterosis for the improvement of this trait. Further, the non-additive gene effects could reduce the progress expected from early generation selection.

Both additive (a) and dominance gene effects (b) are involved in the inheritance of seed dietary fibre, carbohydrate, calcium and phosphorus contents in bean genotypes. Significant b_1_ item revealed that the dominance deviation of gene is predominantly in one direction. Strongly and significant b_2_ item values were noted for these traits, suggesting irregularity of gene distribution. Certainly, the five lines beans probably have different number of genes. In cowpea, the number of gene implied in the inheritance of phosphorus content was ranged from 2.9 to 10.40 while for calcium content, it was situated between 2 and 8.5 (Santos and Boiteux 2015). As observed in the present study, the (b_3_) item was highly significant only for seed carbohydrate and calcium and phosphorus contents suggesting the the existence of inconsistent allelic and non-allelic interaction or dominance effects specific to individual crosses for these traits (Griffing 1956).

Estimates of genetic components by diallel analysis, results revealed that the non-additive components (H1 and H2) was significant and higher than those of additive (*D*) component for seed dietary fibre, carbohydrate, calcium and phosphorus contents, showing more important role of dominance gene effects and clearly asserts the predominance role of both fixable and non-fixable components. Small and non-significant environmental component variation (*E*) for seed dietary fibre, carbohydrate and phosphorus content attested that these characters were strongly under the influence of genetic factors. Likewise, the degree of dominance (H_1_/*D*)^1/2^ was more than unity excepted for seed phosphorus content, and the parents tested had a moderate proportion of dominant gene for all traits, suggesting the over-dominance in the heredity of these traits. However, as the degree of dominance could be biased due to linkage, epistasis or both the over-dominance observed may not be considered as index of true over-dominance (Comstock and Robinson 1952). The values of broad sense heritability were high, except for seed phosphorus content. Therefore, the possibility of obtaining a satisfactory level of these traits through selection based on mean would be successful in improving is evident. Thus, highlighting broad sense heritability trait was least influenced by environment. These findings corroborate those reported by Patrícia et al. (2006), Lenkala et al. (2015), Jemal et al. (2022), and Jiang et al. (2020) showing the high values of broad sense heritability for seed dietary fibre, carbohydrate, seed calcium and phosphorus contents in common bean. The lowest value of narrow sense heritability was noted for seed dietary fibre and carbohydrate, respectively. Therefore, it would be difficult to adopt pedigree method to improve these characters. Similar result was reported by Kevin et al. (2022) for total sugar in common bean. Dalfollo et al. (2014) reported that narrow sense heritability for bean seed calcium content was 64.78%. For seed phosphorus content, the results were in conformity with those of Ribeiro et al. (2011), which obtained the values of broad sense heritability ranging from 59.46 to 82.69% whereas narrow sense heritability oscillated between 21.37 and 65.54%. However, the lowest value of narrow sense heritability estimates confirmed the superiority of over-dominance than additivity and indicates that progress would not be expected with early generation selection for these characters.

The significant results of general combining ability effects indicated that PB, BI, CT and PR genotypes were the best general combiners and were further confirmed by the involvement of these parents in the desirable cross combinations for seed dietary fibre, carbohydrate, calcium and phosphorus contents in bean seeds. The positive GCA of the parental line for a trait indicates its contribution to the high concentration of that trait, while the negative GCA indicates its contribution to low concentration of that trait. Parents with high GCA effects can contribute to the favourable gene flow to offspring and also can provide information about concentration of predominantly additive gene (Xavier et al. 2019). Previous studies showed positive and highly significant GCA effects for dietary fibre in *Psidium guajava* (Lakul and Boonprakob 2019), for carbohydrate in *Cucurbita moschata* (Tamilselvi et al. 2015), and for calcium and phosphorus contents in Maize (Tajwar et al. 2018). Vencovsky and Barriga (1992) mentioned that a high estimate of GCA for a particular parent suggested a higher or lower concentration of favourable alleles. Consequently, high estimates of the general combining ability indicated the superiority of parental lines in mean development, compared to other crosses (Griffing 1956). In perspective, in order to identify more precisely the best parent, the association between higher GCA effects and mean performance are considered to be important criteria for selecting parental line to be used as superior parent in hybridization programme.

Specific combining ability (SCA) analysis showed significant effects for hybrid bean (*p* < 0.01). The crosses PB × BI, CT × PN and BI × PR exhibited positive and highly significant specific combining ability effects for seed dietary fibre, while CT × BI cross had a desirable value of SCA effect of carbohydrate, implying better dietary fibre and carbohydrate yielding hybrids for these traits. CT × BI cross had a desirable value of SCA effect for seed carbohydrate. PB × PN, CT × PR, BI × PN and BI × PR crosses had the best SCA effects for calcium content. PB × CT, CT × BI and CT × PN hybrids were found to be good specific combiners for phosphorus content in common bean.

They further revealed that one good and one poor or even negative general combining parent can be involved in the highly and significant specific combining ability effects of the crosses (Tamilselvi et al. 2015). These crosses could be exploited effectively through heterosis to get desirable recombinants from the segregating population. Tamilselvi et al. (2015) noted significant values of SCA effects for seed dietary fibre and carbohydrate in *Cucurbita moschata*. Strongly and significant specific combining ability (SCA) effects were observed by Tajwar et al. (2018) in Maize. The high degree of genetic complementation between these parents explains their high values for SCA (Machado et al. 2002). SCA effects confirm the consequence of intra-allelic gene interaction (dominance) and inter-allelic gene interaction (epistasis), though the non-significance of the SCA reflects the low contribution of non-additive effects on the expression of these traits.

For dietary fibre, carbohydrate and seed calcium content, the regression line was below the axis origin, intercepting the Wr-axis, indicating the presence of over-dominance in these traits. The estimated regression lines intercepting the Wr-axis above the point of origin confirm the partial dominance in the heredity of seed phosphorus in common bean. The dispersion of parents around the regression line showed that for seed dietary fibre, a higher proportion of recessive genes were expressed in the parents PB, BI and CT, while PR line contained mostly dominant gene. For seed carbohydrate, PR genotype was farther from origin and had an excess of recessive gene, whereas BI genotype was nearer from the point of origin and had the maximum number of dominant gene. The dispersion of parents around the regression line showed that BI genotypes are located further from the origin indicating this parent contains the largest concentration of recessive alleles while PB line was nearer from origin suggesting that mostly dominant alleles for seed calcium content. For seed phosphorus content, the dispersion of parents around the regression line revealed that PR genotype had a maximum recessive gene while PB and CT had mostly dominant gene.

## Conclusion

There were highly significant variable observed among the bean lines for seed dietary fibre, carbohydrate, calcium and phosphorus contents. It suggested that these traits could be improved genetically. The high broad sense heritability observed indicated that the selection for nutritional components at advanced generations would be effective. Utilisation of best general combiners (PB, BI, CT and PR) bean genotypes as the parents produced promising F_1_ hybrids with desirable SCA effects and mean performance for these traits. Additive gene effects were implied in the heredity of seed phosphorus content, and selection in such promising hybrids could be practiced in early segregating generations, and some specific F_1_ hybrids could be identified for hybrid bean production to enhance this trait. For the betterment of improvement bean lines for seed dietary fibre, carbohydrate, calcium and phosphorus contents, full diallel scheme with reciprocal crosses to assess the maternal and paternal effect will be needed. In order to evaluate the stability and the adaptability of bean genotypes for these traits in multiple environments, the study of Genotype x Environmental interaction will be done.
