# Correction to: Diallel analysis of common bean (*Phaseolus vulgaris* L.) genotypes for seed dietary fibre, carbohydrate, calcium and phosphorus contents

**DOI:** 10.1007/s13353-024-00879-8

**Published:** 2024-05-30

**Authors:** Aladji Abatchoua Madi Madi Ibram, Yadji Haman Taidi, Likeng Li Ngue Benoit‑Constant, Noubissié Tchiagam Jean‑Baptiste, Ibrahima Adamou

**Affiliations:** 1 Department of Sciences and Technics of Biological Agriculture, Faculty of Science, University of Ngaoundéré, P.O. Box 454, Ngaoundéré, Cameroon; 2 Department of Biological Sciences, Faculty of Science, University of Ngaoundéré, P.O. Box 454, Ngaoundéré, Cameroon; 3 Genetic and Plant Breeding Unity, Department of Plant Biology, Faculty of Science, University of Yaoundé1, P.O. Box 812, Yaounde, Cameroon

**Correction to** **:** **Journal of Applied Genetics (2024)**

10.1007/s13353-024-00834-7

The original article contains a reference citation error. The following are the corrections.

1. On page 2 and 6, the citation “Sahasakul Amornrat et al. (2022)” should be corrected to “Sahasakul et al. (2022)”.
2. On page 7, the citation “Carovic-Stanko Jerko et al. (2021)” should be corrected to “Carovic-Stanko et al. (2021)”.
3. In the Reference list, Carovic-Stanko Jerko G, Klaudija L, Boris V, Monika MP, Zlatko L, Zlatko Š (2021) should be corrected to “Carovic-Stanko JG, Klaudija L, Boris V, Monika MP, Zlatko L, Zlatko Š (2021)”.
4. In the reference list, de Machado C, F, dos Santos JB, Nunes GH de S, Ramalho MAP, (2002) should be corrected to “Machado de CF, dos Santos JB, Nunes GH de S, Ramalho MAP (2002)”
5. In the reference list, Sahasakul Amornrat YA, Sirinapa T, Pitthaya Parichart WS, Aphinya
6. W, Auytin P, Woorawee I, Piya T, Uthaiwan S (2022) should be corrected to “Sahasakul AYA, Sirinapa T, Pitthaya Parichart WS, Aphinya W, Auytin P, Woorawee I, Piya T, Uthaiwan S (2022)”.

The original article has been corrected.

